# The Impact of Pyroglutamate: *Sulfolobus acidocaldarius* Has a Growth Advantage over *Saccharolobus solfataricus* in Glutamate-Containing Media

**DOI:** 10.1155/2019/3208051

**Published:** 2019-04-24

**Authors:** Anna M. Vetter, Julia Helmecke, Dietmar Schomburg, Meina Neumann-Schaal

**Affiliations:** ^1^Department of Bioinformatics and Biochemistry, Braunschweig Integrated Centre of Systems Biology (BRICS), Technische Universität Braunschweig, Rebenring 56, 38106 Braunschweig, Germany; ^2^Leibniz Institute DSMZ-German Collection of Microorganisms and Cell Cultures, Inhoffenstr. 7B, 38124 Braunschweig, Germany

## Abstract

Microorganisms are well adapted to their habitat but are partially sensitive to toxic metabolites or abiotic compounds secreted by other organisms or chemically formed under the respective environmental conditions. Thermoacidophiles are challenged by pyroglutamate, a lactam that is spontaneously formed by cyclization of glutamate under aerobic thermoacidophilic conditions. It is known that growth of the thermoacidophilic crenarchaeon *Saccharolobus solfataricus* (formerly *Sulfolobus solfataricus*) is completely inhibited by pyroglutamate. In the present study, we investigated the effect of pyroglutamate on the growth of *S. solfataricus* and the closely related crenarchaeon *Sulfolobus acidocaldarius.* In contrast to *S. solfataricus*, *S. acidocaldarius* was successfully cultivated with pyroglutamate as a sole carbon source. Bioinformatical analyses showed that both members of the *Sulfolobaceae* have at least one candidate for a 5-oxoprolinase, which catalyses the ATP-dependent conversion of pyroglutamate to glutamate. In *S. solfataricus*, we observed the intracellular accumulation of pyroglutamate and crude cell extract assays showed a less effective degradation of pyroglutamate. Apparently, *S. acidocaldarius* seems to be less versatile regarding carbohydrates and prefers peptidolytic growth compared to *S. solfataricus*. Concludingly, *S. acidocaldarius* exhibits a more efficient utilization of pyroglutamate and is not inhibited by this compound, making it a better candidate for applications with glutamate-containing media at high temperatures.

## 1. Introduction

Thermoacidophilic organisms are of high interest for biotechnology since the extreme culture conditions offer new possibilities for many different applications. Examples of such thermoacidophilic organisms are found within the *Sulfolobaceae* [1] growing aerobically on different kinds of carbon sources including tryptone and casamino acids [2] at pH 2-3 and temperatures around 75-80°C. However, most thermoacidophiles are restricted by very low biomass yields [3], prohibiting an efficient production of added-value products. One of the reasons is the accumulation of potentially growth-inhibiting ionic compounds of low molecular weight, which was observed during fermenter-based cultivation with *Saccharolobus solfataricus* [3]. One of these compounds has been identified as pyroglutamate, which is spontaneously formed from glutamate and glutamine under high temperature and at low pH [4, 5].

So far, not much is known about the effect of pyroglutamate, neither on eukaryotes nor on prokaryotes. Humans with glutathione synthetase deficiency, leading to pyroglutamate accumulation in blood plasma (pyroglutamic acidemia) and urine (pyroglutamic aciduria), develop chronic metabolic acidosis [6]. Yang and colleagues showed that pyroglutamate, produced from lactic acid bacterial strains, has antimicrobial properties against the investigated gram-negative bacterial strains [7]. Already a pyroglutamate concentration of 0.3% (*w*/*v*) inhibited growth of some *Pseudomonas* and *Enterobacter* strains. Since a stronger inhibitory effect was detectable in liquid media than on agar plates, Yang and colleagues predicted that the antimicrobial effect depends on the undissociated form, which passes the membrane and partially destroys the proton gradient. Pyroglutamate also inhibits the growth of *S. solfataricus* and other thermoacidophiles, e.g., *Metallosphaera sedula* and *Thermoplasma acidophilum* [4, 8]. Park and colleagues showed that supplementation of pyroglutamate in glutamate-containing medium decreases the growth rate of *S. solfataricus* in a concentration range of 3.3-15.5 mM. The growth was decreased by 50% at a concentration of 12.1 mM and was completely abolished at a concentration of 15.5 mM pyroglutamate [4].

It is noteworthy that some hydrolysed proteins used as nutrients in microbial cultures such as casein hydrolysate, and yeast extract [9], contain glutamate as the main amino acid. Biotechnological processes often include complex media containing hydrolysed proteins. These proteins are obtained from animal-derived food waste such as skin or offal and play an important role as cheap carbon sources in biotechnology [8]. Thermophilic organisms are often cultivated in media supplemented with amino acids as carbon sources [3, 10, 11]. However, glutamate is spontaneously converted into pyroglutamate in a pH range of 2 to 3.5 and at temperatures above room temperature [8, 12]. This makes many thermoacidophiles, like, for example, *S. solfataricus*, less suitable for a number of biotechnological approaches due to pyroglutamate-induced growth restriction.

In this study, growth behaviour of two closely related crenarchaea—*S. acidocaldarius* and *S. solfataricus*—on glutamate and pyroglutamate as a sole carbon source was examined. While *S. acidocaldarius* can utilize pyroglutamate even at high concentrations, *S. solfataricus* was inhibited by higher pyroglutamate concentrations even in the presence of other suitable carbon sources. During growth on glutamate and pyroglutamate, *S. solfataricus* exhibited less efficient pyroglutamate metabolization compared to *S. acidocaldarius.* Thus, pyroglutamate is less suitable as a carbon source for *S. solfataricus* since it accumulates inside the cell and inhibits growth at elevated concentrations.

## 2. Materials and Methods

### 2.1. Strain and Growth Conditions

*Sulfolobus acidocaldarius* MW001 [13] and *Saccharolobus solfataricus* P2 [14, 15] were aerobically grown in Brock medium [1] at a pH of 3. The medium of *S. acidocaldarius* MW001 was supplemented with 20 *μ*g/mL uracil. The growth cultures were supplemented by either 24 mM L-glutamate or 24 mM L-pyroglutamate. Both strains were adapted to growth on glutamate starting from a culture grown with casein hydrolysate in a single adaption cycle (5 days). The adaptation to pyroglutamate was performed using glutamate-adapted cells in two cycles (14 days and 4 days). Glycerol stocks of the adapted cells were used for all following experiments.

Four independent cultures were carried out for each condition. The cultures were inoculated (initial OD_600_: 0.05-0.06) with a preculture that was inoculated with an adapted glycerol stock of the same carbon source and were incubated in long neck flasks (500 mL flasks, medium volume 100 mL) at 75°C and 160 rpm (Thermotron, Infors AG, Switzerland). During growth, pH was regulated with 0.5 M H_2_SO_4_. Growth of cells was monitored by measuring optical density at 600 nm (OD_600_).

### 2.2. Quantification of Intracellular and Extracellular Amino Acid Concentration

To determine extracellular concentrations of amino acids, supernatant samples were taken in regular intervals during growth and centrifuged (20,000 ×g, 5 min, RT).

To determine intracellular concentration of amino acids, a culture volume corresponding to 2 mg cell dry weight was harvested at each timepoint by centrifugation (12,000 ×g, 5 min, 4°C). The used OD-biomass correlation was calculated for each carbon source individually. Cell lysis and preparation were performed as described previously [16] with minor modifications: cell pellet was resuspended in 5 mL 0.9% NaCl (*w*/*v*) and washed twice, metabolite extraction was performed with 1/3 volume of methanol (omitting ribitol), deionized water, and chloroform, the polar phase was dried overnight with rotation, and the dried samples were resuspended in 100 *μ*L deionized water. Ammonium was removed from the samples as described previously [17].

Pyroglutamate was analysed after its conversion back to glutamate as described by Macpherson and Slater [18] with slight modifications. 30 *μ*L of the ammonium-free sample was dried with rotation and resolved in 100 *μ*L 6 M HCl followed by an incubation for at least 1.5 h at 95°C and a neutralization step. Afterwards, the sample was dried with rotation and resolved in 30 *μ*L deionized water.

Samples were analysed by HPLC-FLD as described previously [17] with some modifications to focus on glutamate detection: the mobile phase A was changed to 25 mM sodium acetate (pH 6.5) and the gradient was altered for phase B to 3% for 5.3 min, 3-4% within 0.05 min, 4-7% within 4.65 min, 7-15% within 2 min, 15% for 6 min, 15-25% within 0.5 min, 25% for 1 min, 25-30% within 0.5 min, 30-100% within 1 min, and 100% for 2 min.

The pyroglutamate content was calculated as follows: the glutamate content in the sample without conversion was deducted from the glutamate content in the sample after conversion. The resulting concentration corresponds to the pyroglutamate content. The method was confirmed by measuring known concentrations of pyroglutamate and showed a quantitative conversion to glutamate (Figure S1a–b).

### 2.3. Monitoring of Pyroglutamate Conversion in Crude Cell Extract

Just before the cultures reached maximal optical density, a culture volume corresponding to 10 mg cell dry weight was harvested by centrifugation (12,000 ×g, 5 min, 4°C) and resuspended in 500 *μ*L 10 mM HEPES buffer (pH 6.5). Cells were disrupted by sonication thrice with 1 min pulse and 2 min of cooling. Cell debris was removed by centrifugation, and the supernatant was used as crude cell extract. 100 *μ*L of crude cell extract was heated at 65°C for 24 h in 10 mM HEPES buffer containing 2.2 mM L-pyroglutamate and 1 mM MgCl_2_ supplemented with or without 14 mM ATP (pH 6.5) (total volume of 1 mL). As control, 2.2 mM L-pyroglutamate and 14 mM ATP were heated at 65°C for 24 h in 10 mM HEPES buffer supplemented with 1 mM MgCl_2_ (pH 6.5). After heating, proteins were precipitated by chloroform and removed by centrifugation (20,000 ×g, 5 min, 4°C), and the supernatant was analysed for glutamate and pyroglutamate content. Protein concentration of the crude cell extract was determined using the Bicinchoninic acid Protein Assay Kit (Sigma-Aldrich, Germany) following the manufacturer's instructions. Enzymatic activity was calculated based on the difference between the initial pyroglutamate concentration (added pyroglutamate and pyroglutamate of the cell crude extract (CE control)) and the pyroglutamate concentration after 24 h of incubation (CE –/+ ATP) and correlated to a control without cell crude extract (Glp control) and to the protein content of cell crude extract. Quantification of ATP was done using the BacTiter-Glo™ Microbial Cell Viability Assay from Promega (Madison, WI, USA).

### 2.4. Computational Analysis of 5-Oxoprolinase Candidates in *S. acidocaldarius* and *S. solfataricus*

The protein sequences of all 5-oxoprolinase candidates were identified using BLASTp [19] with the protein sequence of the OXP1 gene of *Arabidopsis thaliana* (UniProt: Q9FIZ7) [20]. The sequences of experimentally verified 5-oxoprolinases were obtained from the UniProt database [21], and all sequences were aligned by using Clustal Omega [22]. The genomic context of gene candidates was analysed using the Microbial Genomic Context Viewer [23].

## 3. Results and Discussion

### 3.1. *Sulfolobus acidocaldarius* Exhibits Faster Growth on Glutamate and Uses Pyroglutamate as a Sole Carbon Source

**Figure 1:**
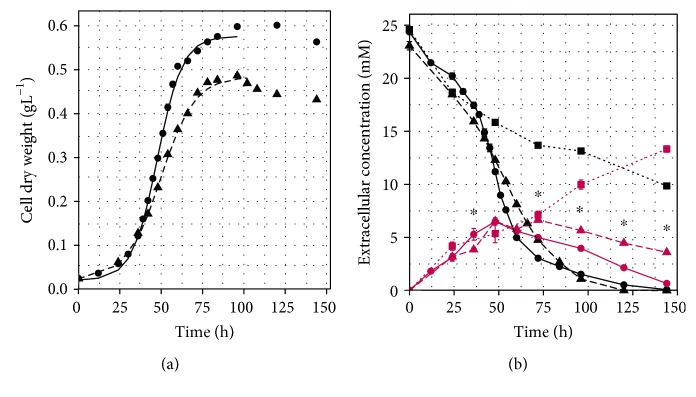
The growth of *Sulfolobus acidocaldarius* and *Saccharolobus solfataricus* on L-glutamate and measured amino acid concentration in the medium. Both strains, *S. acidocaldarius* MW001 (dots) and *S. solfataricus* P2 (triangles), were grown in Brock medium containing 24 mM L-glutamate as a sole carbon source. (a) Growth curve following the cell dry weight per litre of medium *S. acidocaldarius* (dots) and *S. solfataricus* (triangles). Curves were fitted with logistic regression in R. (b) Glutamate (black) and pyroglutamate (red) concentration in supernatant during growth of *S. acidocaldarius* MW001 (dots), *S. solfataricus* P2 (triangles), and an abiotic control (squares). ^∗^Pyroglutamate content differentiated significantly between *S. acidocaldarius* and *S. solfataricus* (Wilcoxon–Mann–Whitney test including Benjamini-Hochberg correction, *p* < 0.05). Values represent the average of four independent cultivations. Error bars represent the standard error between the four experiments.

In the direct comparison of their growth behaviour, *S. acidocaldarius* grew faster and reached a higher maximum cell dry weight than *S. solfataricus* under the same cultivation conditions on glutamate (Figure 1(a); Table 1). Both strains entered the exponential growth phase and the stationary phase at a similar time after inoculation. *S. solfataricus* showed a reduced maximal growth rate and a lower biomass-related substrate uptake rate compared to *S. acidocaldarius* (Table 1). When the cultures reached the stationary phase, the medium contained 1-2 mM glutamate, which was then completely consumed during the stationary phase (Figure 1(b)).

Glutamate is not stable at 75°C and pH 3 [12], leading to pyroglutamate formation (Figure 1(b)) with a reaction rate constant of 0.0066 h^−1^. Therefore, during the cultivation of *Sulfolobaceae* on glutamate, formation of pyroglutamate in the medium was observed. As pyroglutamate was stable under the chosen cultivation conditions (Figure S1b), the decreasing content was the result of consumption. The highest detected concentration of pyroglutamate was almost identical in both cultures, approximately 6.5 mM at 48 h (Figure 1(b)). Afterwards, the pyroglutamate concentration decreased in both culture supernatants. After 48 h, a continuous decrease of glutamate and pyroglutamate was detectable in the supernatant of *S. acidocaldarius* without growth restriction. At the beginning of coutilization, 46% of the initial glutamate concentration was still detectable in supernatant. In the culture of *S. solfataricus*, a continuous pyroglutamate decline was observed after 75 h in the presence of 20% of the initial glutamate concentration. This uptake of pyroglutamate led to no further growth increase. At the end of cultivation, pyroglutamate was almost completely taken up by *S. acidocaldarius* whereas approximately 4 mM pyroglutamate was still detectable in the growth medium of *S. solfataricus*. Combining maximum cell dry weight and residual substrate at OD_max_, both organisms showed a similar yield coefficient (Table 1) at OD_max_.

**Figure 2:**
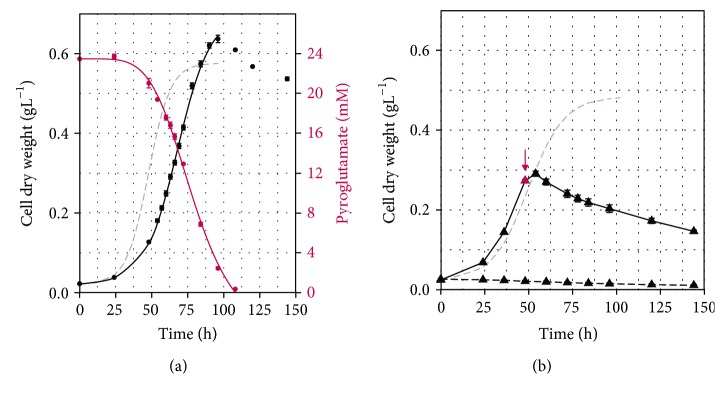
Growth of *Sulfolobus acidocaldarius* and *Saccharolobus solfataricus* on pyroglutamate in comparison to glutamate. (a) Growth of *S. acidocaldarius* MW001 (dots) on 24 mM L-pyroglutamate (black). A fitted curve of cells grown on glutamate was added for better comparison (grey dashed line, see Figure 1(a) for full graph). Pyroglutamate uptake (red). (b) Growth of *S. solfataricus* P2 (triangles) on 24 mM L-glutamate and 14 mM L-pyroglutamate (dashed black line) and on 24 mM L-glutamate (solid black line) spiked with 14 mM L-pyroglutamate at 48 h (red triangle and arrow). A fitted curve of cells grown on glutamate was added for better comparison (grey dashed line, see Figure 1(a) for full graph). Values represent the average of four independent cultivations. Error bars represent the standard error between the four experiments. Curves were fitted with logistic regression in R.

*S. solfataricus* did not grow at all on 24 mM pyroglutamate as a sole carbon source and in the presence of 14 mM pyroglutamate, neither as supplement nor after addition to a growing culture (Figure 2(b)). *S. acidocaldarius* was able to grow on 24 mM pyroglutamate (Figure 2(a)), reaching a maximum cell dry weight of 0.64 g L^−1^ and a maximum growth rate of 0.055 h^−1^ within 96 h of cultivation (Table 1). *S. acidocaldarius* grew slower on pyroglutamate compared to glutamate (Figure 2(a)), but the maximum cell dry weight, maximal substrate uptake rate, and yield coefficient were similar (Table 1).

As stated earlier, we observed a growth-inhibiting effect of pyroglutamate in cultures with *S. solfataricus* (Figure 2(b)). This effect has been previously reported in other studies [4, 8]. Furthermore, we confirmed that the growth-inhibiting effect of pyroglutamate in our experimental set-up is not only due to the loss of available carbon, because its addition to a growing culture can lead to cell death. However, Park and colleagues [4] proposed that pyroglutamate is a competitive inhibitor for glutamate transport in *S. solfataricus*. In contrast, our results show a complete glutamate uptake even in the presence of elevated pyroglutamate concentration which implies that glutamate uptake is rather independent of pyroglutamate occurrence. Moreover, the structures of the two metabolites are very different, thus, we assume that both metabolites may be taken up by different transport systems. For bacteria, it was proposed that pyroglutamate may also passively diffuse through the membrane [7] which may especially occur under acidic cultivation conditions.

### 3.2. Computational Analysis Revealed That Both *Sulfolobaceae* Have at Least One Promising Candidate for Pyroglutamate Degrading Enzymes

To investigate the pyroglutamate degradation capability of both species, we decided to perform a computational analysis of enzyme candidates. The only enzyme found in BRENDA [24] to degrade pyroglutamate was a 5-oxoprolinase (EC 3.5.2.9). This enzyme catalyses the ATP-dependent hydrolysis of pyroglutamate to glutamate. We chose the protein sequence belonging to the OXP1 gene from *Arabidopsis thaliana* as reference because it is the best studied eukaryotic 5-oxoprolinase [20]. We performed a BLAST search to identify gene candidates in *S. acidocaldarius* and *S. solfataricus*. The six identified candidates were Saci_0368, Saci_0369 in *S. acidocaldarius*, and SSO2008, SSO2010, SSO2934, and SSO2936 in *S. solfataricus*. The length of each protein and all sequence identities are shown in Table 2.

A closer look at the genomic context [23] revealed that all genes occurred as gene pairs, coding for two subunits of the protein (Figure S2). A multiple sequence alignment with 5-oxoprolinases from all three domains of life showed that the candidates from *Sulfolobaceae* show high homology to eukaryotic 5-oxoprolinases, with sequence identities between 29 and 40%. We found no satisfying homology to any candidate from Bacteria (maximum 18% sequence identity). However, the 5-oxoprolinases from Eukaryota and Bacteria are encoded by one and three genes, respectively. Contrastingly, the predicted 5-oxoprolinases in *Sulfolobaceae* are encoded by two genes that are in direct genomic context (Figure S3). All seven archaeal 5-oxoprolinases in Swiss-Prot [25] belong to the members of the *Euryarchaeota*. They show high sequence identities with *Sulfolobaceae* candidates between 49 and 54%. The gene candidates SSO2008 and SSO2010 show the highest homology to the 5-oxoprolinase of *Methanocaldococcus jannaschii* (Table 2). Additionally, they consist of two subunits as well. Only members of the family *Methanosarcinaceae* have 5-oxoprolinases that are coded by a single gene. Unfortunately, no crystal structure for any of the 5-oxoprolinases is known; thus, no comparison of the substrate-binding sites was possible.

In summary, we predict that *S. acidocaldarius* has a functional 5-oxoprolinase, consisting of two subunits, and *S. solfataricus* has two copies of this enzyme. Moreover, the 5-oxoprolinase candidates from *Sulfolobaceae* are more homologous to eukaryotes than to bacteria.

For further investigation of the predicted 5-oxoprolinases, we looked at differences in transcription of the gene candidates that could explain the observed phenotypes. Therefore, we analysed published transcriptome data of both members of the *Sulfolobaceae* on different substrates [26–29]. The relative expression levels compared to the median of the whole transcriptome were compared to the presence of glutamate (and accordingly pyroglutamate) in the medium and is shown in Table 3. Although the culture conditions and medium composition differed among the studies, we found a noticeable correlation between the presence of glutamate in the medium and an enhanced expression (up to 5-fold) of the 5-oxoprolinase candidates in *S. acidocaldarius* [27–29]. Contrastingly, the expression of all gene candidates in *S. solfataricus* was remarkably low and did not increase when glutamate was present in the medium [26].

### 3.3. *Saccharolobus solfataricus* Accumulates High Levels of Pyroglutamate and Shows Lower 5-Oxoprolinase Activity

As the growth behaviour showed a strong difference with respect to the usage of the formed pyroglutamate in the later growth state, we further investigated whether both strains metabolise the incorporated pyroglutamate. Therefore, intracellular pyroglutamate and glutamate concentrations during stationary phase were determined.

**Figure 3:**
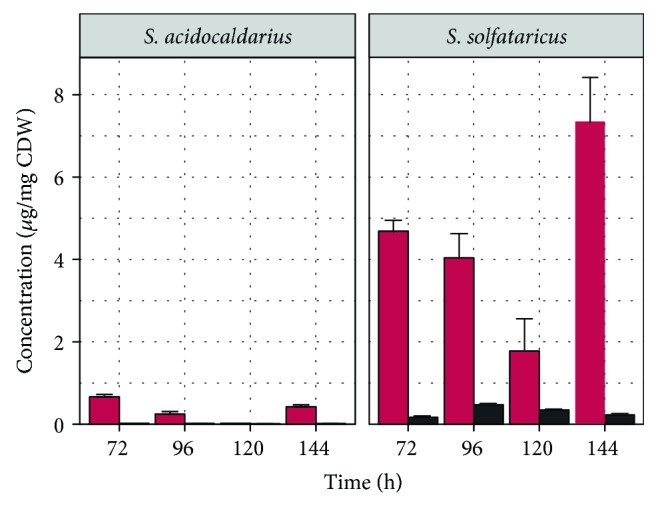
Concentration of intracellular glutamate and pyroglutamate. Glutamate (black) and pyroglutamate (red) concentrations were determined after exponential phase during growth of *Sulfolobus acidocaldarius* MW001 and *Saccharolobus solfataricus* P2 on 24 mM L-glutamate. Values represent the average of four independent cultivations. Error bars represent the standard error between the four experiments.

We observed a large difference in the intracellular content of glutamate and pyroglutamate at the end of cultivation (Figure 3). In *S. acidocaldarius*, both amino acids are present in low levels (less than 1 *μ*g mg^−1^ cell dry weight) whereas the intracellular concentration of glutamate was 10-fold higher and the concentration of pyroglutamate was 17-fold higher in *S. solfataricus*. The intracellular content of pyroglutamate decreased from 72 h to 120 h in both cultures, whereas an accumulation occurred at 144 h. We predict that the cells reached the end of the stationary phase associated with ceased metabolic processes leading to accumulation.

Regarding the differences in intracellular pyroglutamate content, we performed crude cell extract assays to examine differences in the enzymatic activity based on pyroglutamate consumption. Additionally, we detected the formation of glutamate in this assay (Table S1). However, a quantitative evaluation of glutamate production is hampered by a possible further conversion of glutamate by other enzymes in the crude cell extract.

**Figure 4:**
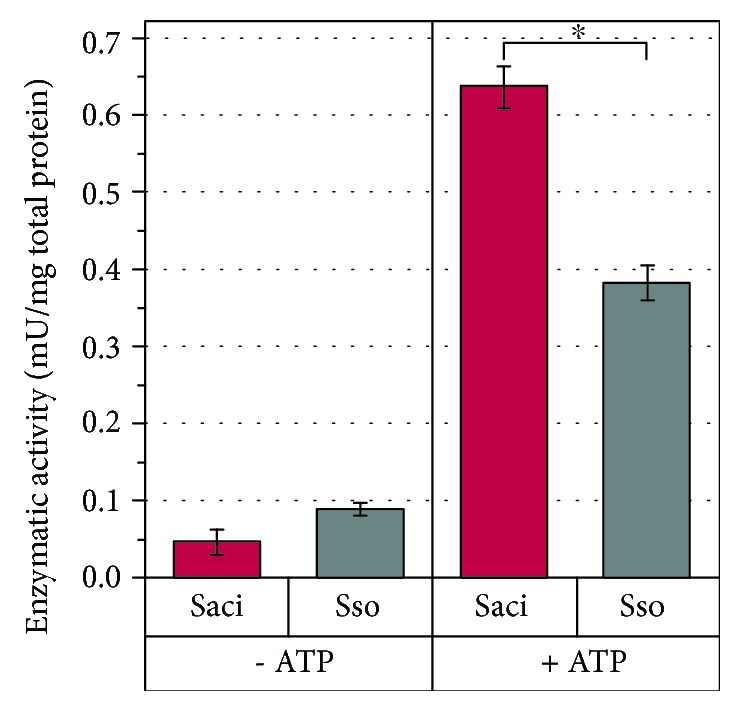
Enzymatic activity in crude cell extracts of *Sulfolobus acidocaldarius* and *Saccharolobus solfataricus.* Enzymatic activity was calculated for crude cell extracts of *S. acidocaldarius* MW001 (Saci, red) and *S. solfataricus* P2 (Sso, grey) supplemented with 2.2 mM L-pyroglutamate and in the presence and absence of 14 mM ATP (+ATP/-ATP) at 65°C for 24 h. ^∗^Pyroglutamate content differentiated significantly between *S. acidocaldarius* and *S. solfataricus* (Wilcoxon–Mann–Whitney test including Benjamini-Hochberg correction, *p* < 0.05). Enzymatic activity was calculated based on the pyroglutamate consumption compared to a control without crude cell extract and correlated to the total protein content. One U is defined as the conversion of one *μ*mol pyroglutamate per minute. A sufficient residual ATP content was confirmed after 24 h (data not shown). Values represent the average of four independent cultivations. Error bars represent the standard error between the four experiments.

We detected 5-oxoprolinase activity in crude cell extracts of both strains (Figure 4). The enzymatic activity was strongly enhanced in the presence of ATP and was 1.7-fold higher in the crude cell extract of *S. acidocaldarius* compared to *S. solfataricus*. A control without cell crude extracts did not show a spontaneous degradation of pyroglutamate in the presence of ATP (Table S1).

In summary, in the cells of *S. acidocaldarius*, glutamate and pyroglutamate were both detected in substantially lower concentrations than in *S. solfataricus*, indicating a rapid conversion of the carbon source for metabolic processes. The data suggest that pyroglutamate is a higher burden for *S. solfataricus* than for *S. acidocaldarius* since it accumulates in the cells to more than 0.7% (*w*/*w*) of the cell dry weight. Taken into account that microorganisms typically contain approximately 3% (*w*/*w*) low molecular weight metabolites [30], this indicates a strong accumulation. We see a cooccurrence of enhanced intracellular pyroglutamate levels and growth inhibition in *S. solfataricus*. In this context, the previously stated role of pyroglutamate as a classical protonophore with cycles of passive uptake in the protonated and an active export in the deprotonated form with a comparable low intracellular concentration [4, 8] is not supported by our data. We showed a strong intracellular accumulation in *S. solfataricus* indicating an active energy-demanding import without subsequent energy-producing catabolic reactions. Inside the cell, the accumulation of an acid may lead to severe alterations of the metabolism, e.g., by decreased protein stability or inhibitory effects, and may serve as an explanation for the toxic effect. Finally, performed crude cell extract assays revealed a more effective conversion of pyroglutamate in *S. acidocaldarius*. Therefore, *S. acidocaldarius* took advantage of using pyroglutamate as a carbon source for anabolic processes and respiration.

## 4. Conclusion

In this study, we examined the effect of pyroglutamate on the thermoacidophilic crenarchaea *Sulfolobus acidocaldarius* and *Saccharolobus solfataricus*. During glutamate cultivation, we observed spontaneous pyroglutamate formation from glutamate and improved growth of *S. acidocaldarius.* Analysis of intracellular glutamate and pyroglutamate concentrations shows that *S. solfataricus* accumulates much higher levels of both amino acids in the cytoplasm upon reaching the steady state, while the levels in *S. acidocaldarius* remain low. A computational analysis of several gene candidates revealed that both strains contain gene candidates for 5-oxoprolinases capable of degrading pyroglutamate, but we detected a lower 5-oxoprolinase activity in crude cell extracts of *S. solfataricus*. Our data imply that pyroglutamate is a higher burden for *S. solfataricus*, because it leads to complete growth inhibition at higher concentrations. This is further supported by the fact that only *S. acidocaldarius* grew on pyroglutamate as a sole carbon source.

To our knowledge, the growth of thermoacidophilic archaea on pyroglutamate as a sole carbon source has not been reported before. This ability of *S. acidocaldarius* makes it into a highly suitable candidate for high temperature biotechnological applications, including the degradation of glutamate-rich media without any negative effect of spontaneously formed pyroglutamate.

## Figures and Tables

**Table 1:** Growth parameters of *Sulfolobus acidocaldarius* and *Saccharolobus solfataricus*. Maximum growth rate *μ* (h^−1^), maximum cell dry weight CDW_max_ (g L^−1^), maximal substrate (glutamate and pyroglutamate) uptake rate qS_max_ (mmol g_CDW_^−1^ h^−1^), and yield coefficient Y at OD_max_ (g_CDW_/mol of amino acid carbon) of *S. acidocaldarius* MW001 and *S. solfataricus* P2 grown on L-glutamate (Glu) or L-pyroglutamate (Glp) (each 24 mM). Values represent the average of four independent experiments. Errors represent the standard deviation.

	Amino acid	*μ*_max_ (h^−1^)	CDW_max_ (g L^−1^)	qS_max_ (mmol g_CDW_^−1^ h^−1^)	Y (g_CDW_ mol_C_^−1^)
*S. acidocaldarius*	Glu	0.078 ± 0.001	0.60 ± 0.01	1.86 ± 0.14	5.70 ± 0.37
*S. solfataricus*	Glu	0.049 ± 0.001	0.49 ± 0.01	1.13 ± 0.09	6.04 ± 0.11
*S. acidocaldarius*	Glp	0.055 ± 0.001	0.64 ± 0.02	1.63 ± 0.01	5.84 ± 0.09
*S. solfataricus*	Glp	nd	nd	nd	nd

nd: no growth during the cultivation period of 22 d detected.

**Table 2:** Identification of putative 5-oxoprolinases. Percent identity matrix, BLAST e-values, and protein length of all 5-oxoprolinase protein candidates.

	Length	Saci_0368	Saci_0369	SSO2010	SSO2008	SSO2934	SSO2936
Saci_0368	505	100	0	64.9	0	62.3	0
		0.0*E* + 00		0.0*E* + 00		0.0*E* + 00	

Saci_0369	640	0	100	0	61.8	0	58.7
			0.0*E* + 00		0.0*E* + 00		0.0*E* + 00

SSO2010	510	64.9	0	100	0	79.4	0
		0.0*E* + 00		0.0*E* + 00		0.0*E* + 00	

SSO2008	644	0	61.8	0	100	0	77.8
			0.0*E* + 00		0.0*E* + 00		0.0*E* + 00

SSO2934	513	62.3	0	79.4	0	100	0
		0.0*E* + 00		0.0*E* + 00		0.0*E* + 00	

SSO2936	650	0	58.7	0	77.8	0	100
			0.0*E* + 00		0.0*E* + 00		0.0*E* + 00

METJA_963	563	49.9	0	53.17	0	49.31	0
		8.9*E*-162		1.6*E*-173		2.4*E*-168	

METJA_964	680	0	47.89	0	51.24	0	49.92
			0.0*E* + 00		0.0*E* + 00		0.0*E* + 00

ARATH	1266	34.8	31.2	38.7	32.8	37.5	31.5
		1.8*E*-95	8.5*E*-63	4.7*E*-110	1.8*E*-75	7.7*E*-101	1.1*E*-69

HUMAN	1288	35.6	32.7	39.5	32.8	35.8	31.1
		1.9*E*-85	9.8*E*-60	7.0*E*-107	8.0*E*-64	5.3*E*-95	2.5*E*-58

MOUSE	1288	35.4	32.7	38.5	33	35.6	30.9
		1.2*E*-79	1.6*E*-56	6.4*E*-97	1.4*E*-62	1.7*E*-87	2.3*E*-56

Abbreviations and UniProt accessions: ARATH = *Arabidopsis thaliana* (Q9FIZ7), HUMAN = *Homo sapiens* (O14841), MOUSE = *Mus musculus* (Q8K010), METJA = *Methanocaldococcus jannaschii* (Q58373 & Q58373).

**Table 3:** Transcript levels of 5-oxoprolinase candidates from *S. acidocaldarius* and *S. solfataricus*. The expression levels are the RPKM values normalized by the median RPKM of the whole transcriptome. The source of all published transcriptome data can be found under reference.

Organism	Locus	Presence of glutamate in medium	Expression level compared to median	Reference
*S. acidocaldarius*	Saci_0368	+	4.57	[28]
	Saci_0369	+	6.76	[28]
	Saci_0368	+	3.00	[27]
	Saci_0369	+	3.43	[27]
	Saci_0368	—	1.45	[29]
	Saci_0369	—	1.16	[29]

*S. solfataricus*	SSO2008	+	1.32	[26]
	SSO2010	+	0.83	[26]
	SSO2936	+	0.51	[26]
	SSO2934	+	0.40	[26]
	SSO2008	—	1.24	[26]
	SSO2010	—	0.69	[26]
	SSO2936	—	0.87	[26]
	SSO2934	—	0.81	[26]

## Data Availability

The data used to support the findings of this study are included within the article and the supplementary information files.

## Supplementary Materials

Supplementary Materials Figure S1: evaluation of pyroglutamate measurement procedure. (a) Conformity of appointed and measured pyroglutamate concentrations. Pyroglutamate content in supernatant at a timepoint 0 h and 24 h of the S. acidocaldarius growth curve supplemented with 24 mM pyroglutamate (see Figure 2(a)). (b) Pyroglutamate stability at thermoacidophilic conditions. 22 mM pyroglutamate was cultivated in Brock Medium at 75°C and pH 3 for 144 h. Values represent the average of four independent cultivations, and error bars represent the standard error. Figure S2: genomic context of all 5-oxoprolinase candidates from *Sulfolobus acidocaldarius* and *Saccharolobus solfataricus*. This figure was created using the Microbial Genomic Context Viewer. Figure S3: fingerprint of the multiple sequence alignment of 5-oxoprolinases from all three domains of life. Blue sequence names indicate the 5-oxoprolinase candidates from *Sulfolobus acidocaldarius* and *Saccharolobus solfataricus*. Light grey shadings indicate the nonconserved regions, blue shading shows over 30% conservation, and red shading indicates similar regions. Organisms: BACSU = *Bacillus subtilis* 168, ECOLI = *Escherichia coli* K12, METJA = *Methanocaldococcus jannaschii* JAL-1, SULAC = *Sulfolobus acidocaldarius* DSM 639, SULSO = *Saccharolobus solfataricus* P2, METBF = *Methanosarcina barkeri* DSM 804, ARATH = *Arabidopsis thaliana*, and HUMAN = *Homo sapiens*. Table S1: content of glutamate and pyroglutamate in crude cell extract assay of *Sulfolobus acidocaldarius* and *Saccharolobus solfataricus*. Crude cell extracts (CE) of *S. acidocaldarius* MW001 (Saci) and *S. solfataricus* P2 (Sso) were incubated with 2.2 mM L-pyroglutamate (Glp) in presence and absence of 14 mM ATP (+ATP/-ATP) at 65°C for 24 h. Afterwards, glutamate (Glu) and pyroglutamate content were determined. Values represent the average of four independent cultivations. Errors represent the standard error between the four experiments.
