# Supplementary material for: The Impact of Pyroglutamate: Sulfolobus acidocaldarius Has a Growth Advantage over Saccharolobus solfataricus in Glutamate-Containing Media
Source: Archaea. 2019 Apr 24;2019:3208051. doi: 10.1155/2019/3208051 (PMC6507225; doi:10.1155/2019/3208051)
Supplement: Supplementary Materials — Figure S1: evaluation of pyroglutamate measurement procedure. (a) Conformity of appointed and measured pyroglutamate concentrations. Pyroglutamate content in supernatant at a timepoint 0 h and 24 h of the S. acidocaldarius growth curve supplemented with 24 mM pyroglutamate (see Figure 2(a)). (b) Pyroglutamate stability at thermoacidophilic conditions. 22 mM pyroglutamate was cultivated in Brock Medium at 75°C and pH 3 for 144 h. Values represent the average of four independent cultivations, and error bars represent the standard error. Figure S2: genomic context of all 5-oxoprolinase candidates from Sulfolobus acidocaldarius and Saccharolobus solfataricus. This figure was created using the Microbial Genomic Context Viewer. Figure S3: fingerprint of the multiple sequence alignment of 5-oxoprolinases from all three domains of life. Blue sequence names indicate the 5-oxoprolinase candidates from Sulfolobus acidocaldarius and Saccharolobus solfataricus. Light grey shadings indicate the nonconserved regions, blue shading shows over 30% conservation, and red shading indicates similar regions. Organisms: BACSU = Bacillus subtilis 168, ECOLI = Escherichia coli K12, METJA = Methanocaldococcus jannaschii JAL-1, SULAC = Sulfolobus acidocaldarius DSM 639, SULSO = Saccharolobus solfataricus P2, METBF = Methanosarcina barkeri DSM 804, ARATH = Arabidopsis thaliana, and HUMAN = Homo sapiens. Table S1: content of glutamate and pyroglutamate in crude cell extract assay of Sulfolobus acidocaldarius and Saccharolobus solfataricus. Crude cell extracts (CE) of S. acidocaldarius MW001 (Saci) and S. solfataricus P2 (Sso) were incubated with 2.2 mM L-pyroglutamate (Glp) in presence and absence of 14 mM ATP (+ATP/-ATP) at 65°C for 24 h. Afterwards, glutamate (Glu) and pyroglutamate content were determined. Values represent the average of four independent cultivations. Errors represent the standard error between the four experiments. [file 3208051.f1.pdf]

Supplementary Material

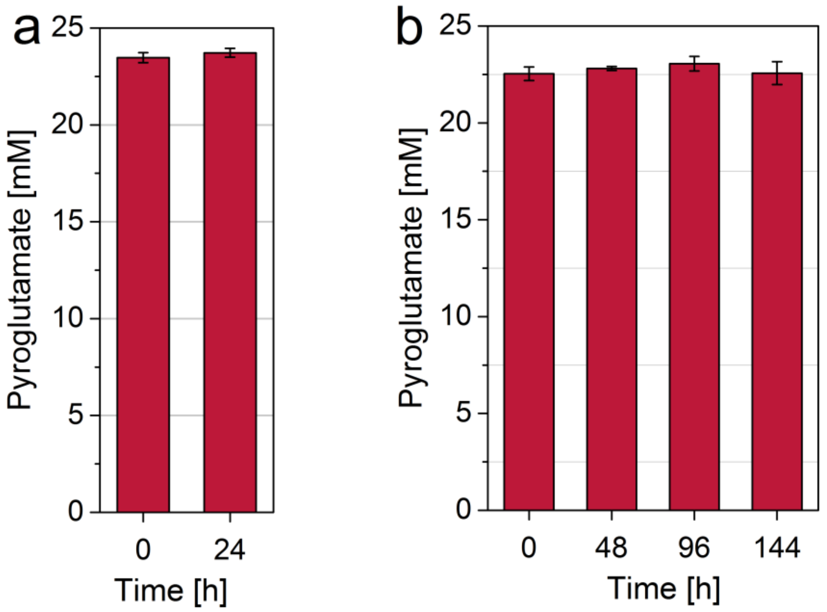

Figure S1: Evaluation of pyroglutamate measurement procedure. (a) Conformity of appointed and measured pyroglutamate concentrations. Pyroglutamate content in supernatant at time-point 0 h and 24 h of the *S. acidocaldarius* growth curve supplemented with 24 mM pyroglutamate (see Figure 2a). **(b)** Pyroglutamate stability at thermoacidophilic conditions. 22 mM pyroglutamate were cultivated in Brock Medium at 75°C and pH 3 for 144 h. Values represent the average of four independent cultivations and error bars represent the standard error.

Table S1: Content of glutamate and pyroglutamate in crude cell extract assay of *Sulfolobus acidocaldarius* and *Saccharolobus solfataricus*. Crude cell extracts (CE) of *S. acidocaldarius* MW001 (Saci) and *S. solfataricus* P2 (Sso) was incubated with 2.2 mM L-pyroglutamate (Glp) in presence and absence of 14 mM ATP (-ATP/+ATP) at 65°C for 24 h. Afterwards glutamate (Glu) and pyroglutamate content were determined. Values represent the average of four independent cultivations. Errors represent the standard error between the four experiments.

Name	Crude cell extract	Supplementation		Content after incubation	
		ATP	Glp	Glu [mM]	Glp [mM]
Glp control	-	+	+	0.01 ± 0.00	2.20 ± 0.02
CE control	*S. acidocaldarius*	-	-	0.23 ± 0.01	0.20 ± 0.02
	S. solfataricus	-	-	0.18 ± 0.01	0.26 ± 0.02
CE – ATP	*S. acidocaldarius*	-	+	0.20 ± 0.01	2.34 ± 0.05
	S. solfataricus	-	+	0.14 ± 0.01	2.34 ± 0.03
CE + ATP	*S. acidocaldarius*	+	+	1.11 ± 0.03	1.53 ± 0.05
	S. solfataricus	+	+	0.67 ± 0.02	1.92 ± 0.04

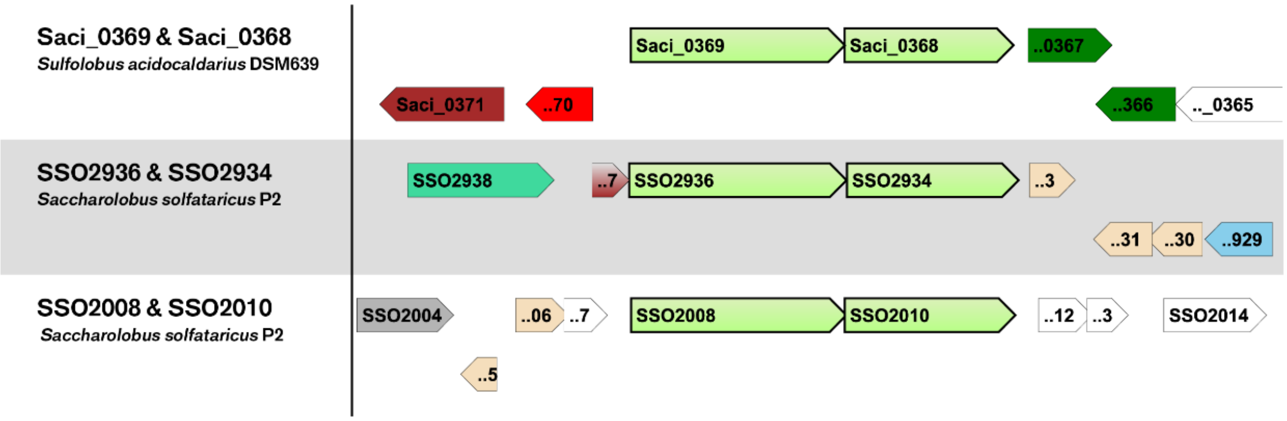

Figure S2: Genomic context of all 5-oxoprolinase candidates from *Sulfolobus acidocaldarius* and *Saccharolobus solfataricus*. This figure was created using the Microbial Genomic Context Viewer.

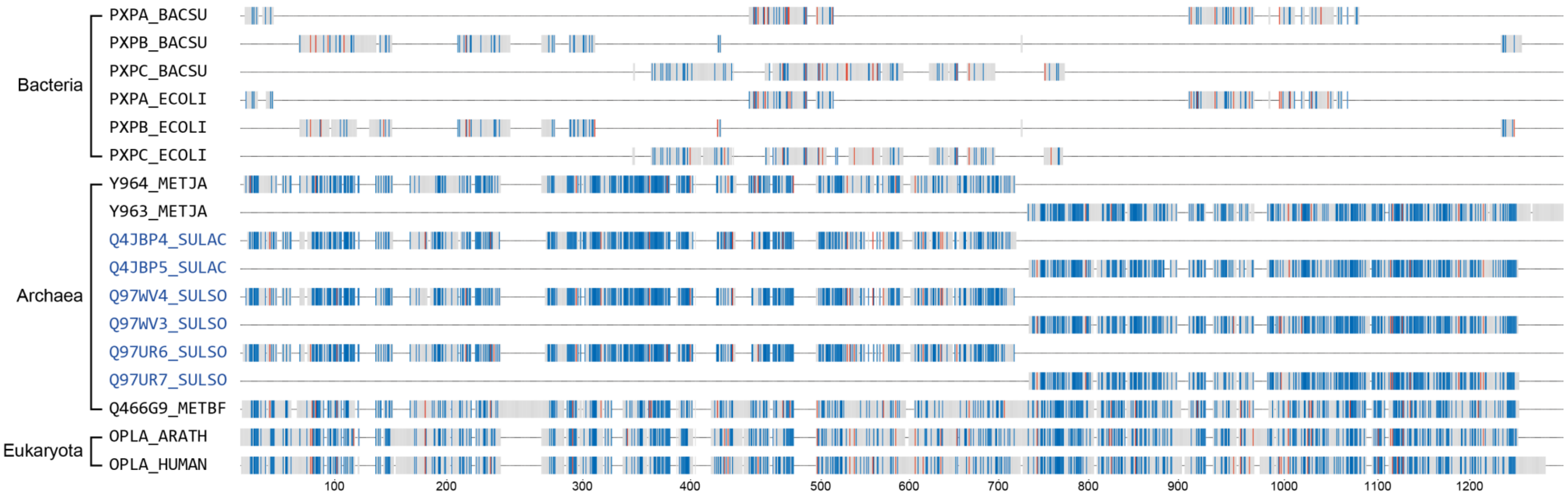

Figure S2: Fingerprint of the multiple sequence alignment of 5-oxoprolinases from all three domains of life. Blue sequence names indicate the 5-oxoprolinase candidates from *Sulfolobus acidocaldarius* and *Saccharolobus solfataricus*. Light gray shadings indicate non-conserved regions, blue shading shows over 30% conservation, and red shading indicates similar regions. Organisms: BACSU = *Bacillus subtilis* 168, ECOLI = *Escherichia coli* K12, METJA = *Methanocaldococcus jannaschii* JAL-1, SULAC = *Sulfolobus acidocaldarius* DSM 639, SULSO = *Saccharolobus solfataricus* P2, METBF = *Methanosarcina barkeri* DSM 804, ARATH = *Arabidopsis thaliana*, HUMAN = *Homo sapiens*.
